# Associations Between Breast Milk Energy Intake, Infant Sleep Duration and Time Until Next Feed

**DOI:** 10.3390/clockssleep8030056

**Published:** 2026-09-08

**Authors:** Kaitlyn A. Rae, K. Swaminathan Iyer, Nicolas L. Taylor, Sharon L. Perrella, Jacki L. McEachran, Zoya Gridneva, Ashleigh H. Warden, Ching Tat Lai, Philip Vlaskovsky, Donna T. Geddes

**Affiliations:** 1 School of Molecular Sciences, The University of Western Australia, Crawley, WA 6009, Australia; kaitlyn.rae@research.uwa.edu.au (K.A.R.); swaminatha.iyer@uwa.edu.au (K.S.I.); nicolas.taylor@uwa.edu.au (N.L.T.); zoya.gridneva@uwa.edu.au (Z.G.);; 2 ARC Training Centre for Next-Gen Technologies in Biomedical Analysis, The University of Western Australia, Crawley, WA 6009, Australia; 3 The Centre for Human Lactation Research and Translation, The University of Western Australia, Crawley, WA 6009, Australia; 4 ABREAST Network, Perth, WA 6000, Australia; 5 School of Physics, Mathematics and Computing, The University of Western Australia, Crawley, WA 6009, Australia

**Keywords:** lactation, breastfeeding, human milk, breast milk, infant sleep, energy intake, macronutrients

## Abstract

Background: Sleep in early infancy supports neurodevelopment, yet the influence of breast milk composition on sleep duration remains unclear. Objective: To determine whether breast milk energy intake is associated with the duration and timing of subsequent infant sleep episodes. Methods: Thirty-four healthy, fully breastfeeding mother–infant dyads (infant age: 3.7 ± 1.3 months) recorded 24-h infant feeding and sleep data, with feed volumes measured by pre- and post-feed test-weighing. Milk samples were collected and analysed for macronutrient concentrations to calculate energy intake per feed. Linear mixed-effects models were used to examine whether energy intake from the preceding feed was associated with subsequent sleep duration. Additional models evaluated lagged and cumulative energy intake, and mixed-effects modelling was used for associations between feed energy and time until the next feed. Results: Intake for 24-h infant breast milk was 851.8 ± 194.2 mL and sleep duration was 15.5 ± 3.4 h. The mean energy density of the feeds was 674.7 ± 140.7 kcal/L. Energy intake for the breastfeed preceding a sleep episode was not associated with sleep duration (*β* = 4.13 min; 95% CI −0.89 to 9.15; *p* = 0.106). Energy intake from lagged feeds and cumulative energy intake across the three preceding breastfeeds were also not associated with sleep duration (*p* = 0.171 and *p* = 0.290 respectively) nor the time until the next breastfeed (*β* = −0.025; 95% CI −6.2 to 1.4; *p* = 0.212). Conclusions: In healthy breastfed infants, the energetic content of breast milk feeds was not associated with subsequent sleep duration or the time until the next feed. These findings suggest that infant sleep–wake behaviour and feeding patterns were not associated with short-term variation in breast milk energy intake. Other factors, including circadian and developmental processes, are likely to contribute to infant sleep behaviour.

## 1. Introduction

Sleep and feeding are closely linked during early infancy, with feeding episodes shaping the timing and structure of sleep across the day and night [1,2,3]. While sleep becomes increasingly consolidated across the first year of life, disrupted infant sleep remains a common concern for caregivers and is commonly managed through feeding-based strategies [4]. In the context of breastfeeding, these strategies may extend beyond feeding frequency or volume to focus on the perceived energy content of feeds, reflecting widespread beliefs that higher-energy breast milk (i.e., more “hind milk”) promotes longer infant sleep duration [5,6,7].

Breast milk is a dynamic biological fluid, and its macronutrient composition varies within and between feeds, across the day, and between individuals, particularly fat content [8,9]. Fat, protein, and lactose contribute differently to milk energy density and digestion, with potential implications for infant satiety and feeding behaviour [10,11]. However, macronutrient concentrations alone do not reflect the quantity consumed by the infant. Accurate estimation of infant fat and energy intake requires both representative milk sampling and precise quantification of breast milk intake, which varies substantially between infants and feeds [12]. Supporting this, significant time-dependent relationships have been found for breast milk feed intake, whereas no associations have been observed for lactose and protein concentrations. This suggests that milk composition without intake quantification may provide limited insight into infant feeding and sleep behaviours [13].

Despite increasing interest in infant sleep and feeding dynamics, existing studies have largely compared feeding methods (breastfeeding versus formula feeding) or feeding frequency and have relied predominantly on caregiver-reported feeding and sleep outcomes [14,15,16,17]. Recent advances using test-weighing have enabled the accurate measurement of breast milk intake at the level of individual feeds, allowing closer examination of feed–sleep dynamics [10,18]. Consequently, it remains unclear whether higher breast milk energy intake is associated with infant sleep duration, or whether its effects are more closely related to the timing of subsequent feeding episodes [19].

This study examined whether the energy and macronutrient composition of breast milk were associated with infant sleep duration and time to the next feed. Using 24-h feed-level measurements of breast milk composition, breast milk feed intake, and parent-reported recorded sleep episodes, we sought to determine whether higher milk energy intake is associated with longer subsequent sleep duration or extended feeding intervals, independent of infant age and the time of day of the sleep episode (daytime versus nighttime).

## 2. Results

### 2.1. Participant Characteristics

Mothers had a mean age of 34.1 ± 4.7 years and pre-pregnancy BMI of 26.5 ± 4.8 kg/m^2^ (*n* = 34). Most mothers (58.8%) were primiparous, and the majority identified as Australian (79.4%). Mean birth gestation was 39.5 ± 1.4 weeks, with a birth weight of 3482.1 ± 384.6 g. Female infants represented 55.9% of the cohort (Table 1).

Across the recording period, mean study duration was 26.1 ± 2.0 h and infants slept for a cumulative 15.5 ± 3.4 h. Infant age at time of sampling was 3.7 ± 1.3 months, with a total 24-h breast milk intake of 851.8 ± 194.2 mL. Mean maternal breast milk production was 873.2 ± 191.2 mL. All included mothers had breast milk production volumes above the established threshold for adequate milk supply (>600 mL/24 h) [16,20].

**Figure 1:**
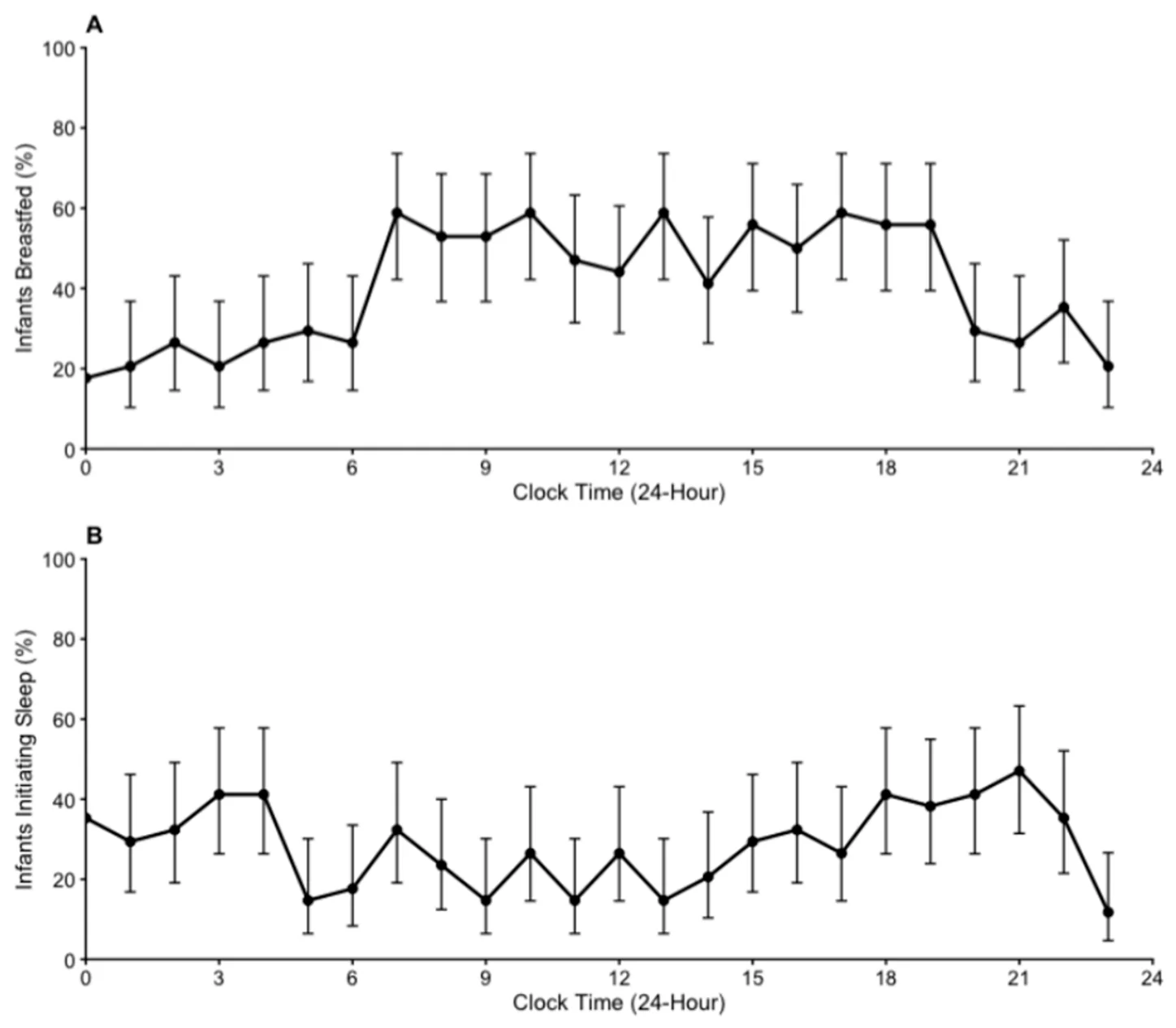
Twenty-four-hour patterns of breastfeeding and infant sleep. (**A**) Percentage of infants with at least one breastfeeding episode beginning within each clock hour. Data represents 509 breastfeeding episodes with recorded start times from 34 infants. (**B**) Percentage of infants with at least one sleep episode beginning within each clock hour. Data represents 237 sleep episodes with recorded start times from 34 infants. Clock time represents the recorded start time of each breastfeeding or sleep episode. Points represent percentages and error bars represent 95% confidence intervals.

Infants averaged 16.1 ± 4.9 breastfeeds and 7.2 ± 1.6 sleep episodes over the 24 h (Table 2). Breastfeeding and sleep episodes occurred throughout the 24-h recording period, with the hourly distribution of breastfeeding and sleep episode onset presented in Figure 1.

### 2.2. Breast Milk Macronutrient Concentrations and Intakes

Mean fat concentration was 38.5 ± 15.4 g/L, corresponding to a daily fat intake of 32.5 ± 14.5 g/day. Mean protein and lactose concentrations were 9.1 ± 3.1 g/L and 73.0 ± 15.2 g/L, with corresponding daily intakes of 7.6 ± 2.5 g/day and 62.9 ± 17.9 g/day respectively. Total daily energy intake derived from breast milk was 570.8 ± 154.0 kcal/day, calculated from a mean energy density of 674.7 ± 140.7 kcal/L. Fat contributed the largest proportion of total energy intake (49.8 ± 12.1%), followed by lactose (44.6 ± 11.4%). Protein contributed the smallest proportion of total energy intake (5.6 ± 2.2%) (Table 3).

**Figure 2:**
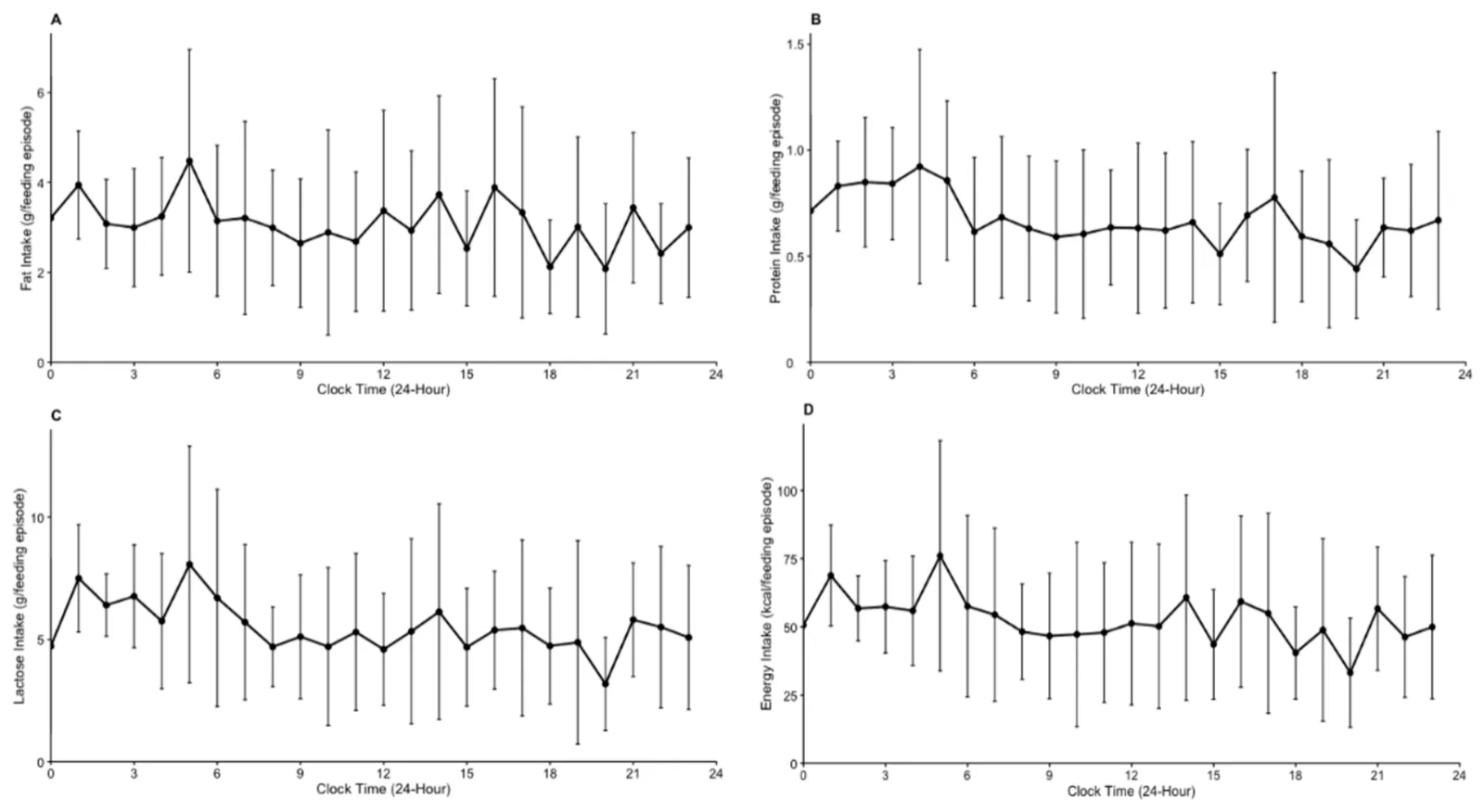
Twenty-four-hour variation in macronutrient and energy intake per breastfeeding episode. (**A**) Fat intake (g/feeding episode), (**B**) protein intake (g/feeding episode), (**C**) lactose intake (g/feeding episode), and (**D**) energy intake (kcal/feeding episode). Points represent mean intake within each hourly interval and error bars represent ±1 SD. Where an infant contributed more than one breastfeeding episode within the same hourly interval, intake was first averaged within the infant so that each infant contributed one value per hourly interval. Hourly means and SDs were subsequently calculated across infants.

The 24-h variation in fat, protein, lactose and energy intake per breastfeeding episode is presented in Figure 2.

### 2.3. Breast Milk Energy Intake and Subsequent Sleep Duration

Of the 249 valid sleep episodes recorded, 237 (95.2%) had an identifiable preceding feed and were included in the analyses. Twelve sleep episodes (4.8%) were excluded because no preceding feed could be identified. The excluded episodes had a mean duration of 136 ± 154 min (median 90 min; interquartile range 36.8–170 min), which was comparable to the included episodes (130 ± 124 min; median 90 min). Among the 237 sleep episodes included in the primary analysis, the median interval between the immediately preceding recorded feed and subsequent sleep onset was 120 min (IQR: 47–228 min), with 22.8% of intervals exceeding 4 h.

Across all three models, energy intake was not associated with subsequent infant sleep duration. In Model 1, energy intake from the preceding feed was not associated with subsequent sleep duration (*β* = 4.13 min per 10 kcal, 95% CI −0.89 to 9.15, *p* = 0.106). In Model 2, which included energy from the current feed (t) and the two preceding feeds (t^−1^, t^−2^), energy intake was not associated with sleep duration (energy t: *β* = 3.52 min per 10 kcal, *p* = 0.171; energy t^−1^: *β* = 2.28 min, *p* = 0.412; energy t^−2^: *β* = −1.87 min, *p* = 0.518). Similarly, cumulative energy intake across the three most recent feeds was not associated with subsequent sleep duration in Model 3 (*β* = 1.37 min per 10 kcal, 95% CI −1.18 to 3.91, *p* = 0.290). Covariates including infant age, time of day of the sleep episode, birth weight, and 24 h milk production were not significantly associated with sleep duration in the adjusted models (Table 4). The unadjusted analyses produced results consistent with the adjusted models, with no significant associations between breast milk energy intake and subsequent sleep duration (Supplementary Table S1).

To further examine associations between nutritional intake and sleep across the complete 24-h period, within-subject analyses were conducted using hourly measures of intake and sleep duration. The analysis included 816 hourly observations from 34 infants. No significant associations were observed between hourly sleep duration and energy intake (*β* = −0.152 min per 10 kcal, 95% CI −0.433 to 0.130, *p* = 0.290), fat intake (*β* = −0.274 min/g, 95% CI −0.726 to 0.178, *p* = 0.234), protein intake (*β* = −1.124 min/g, 95% CI −3.249 to 1.002, *p* = 0.300), lactose intake (*β* = −0.117 min/g, 95% CI −0.395 to 0.161, *p* = 0.407), or milk volume (*β* = −1.044 min per 100 mL, 95% CI −3.014 to 0.926, *p* = 0.299) (Table 5).

In a post hoc sensitivity analysis restricted to sleep episodes occurring within 4 h of the preceding feed (183 sleep episodes; 34 infants), preceding-feed energy intake remained unassociated with subsequent sleep duration (*β* = 3.24 min per 10 kcal, 95% CI −1.88 to 8.37; *p* = 0.213). An additional sensitivity analysis was performed after restricting the cohort to infants aged 2–6 months (28 infants; 195 sleep episodes). Excluding infants younger than 2 months and older than 6 months did not alter the results. No significant associations were observed between feed energy intake and subsequent sleep duration in any of the three models (Supplementary Table S2).

### 2.4. Breast Milk Energy Intake and Time Until Next Feed

To assess whether feed energy intake was associated with subsequent feeding behaviour, inter-feed intervals were calculated as the time between consecutive feeds within each infant. In mixed-effects models accounting for repeated feeding episodes and temporal autocorrelation, feed energy intake was not associated with time until the next feed (*β* = −2.4% per 10 kcal, 95% CI −6.2 to 1.4, *p* = 0.212). Infant age, time of day of the sleep episode, birth weight, and 24-h milk production were also not significantly associated with inter-feed interval duration (Table 6).

## 3. Discussion

This study examined whether the energy and macronutrient composition of breast milk was associated with subsequent infant sleep duration using time-ordered 24-h feeding and sleep data. Breast milk energy intake was not associated with infant sleep duration, either from the immediately preceding feed or cumulatively across multiple feeds. Additionally, energy intake was not associated with the time until the next feed. These results demonstrate no association between short-term variation in feed energy intake and infant sleep duration or feeding behaviour, while other biological and developmental factors may contribute to these outcomes.

Across the 24-h recording period, infants in this cohort exhibited feeding and sleep patterns typical of early infancy, with frequent feeding episodes distributed across day and night and fragmented ultradian sleep architecture characterised by multiple sleep episodes [1,21] (Table 2; Figure 1). Total daily intake and maternal milk production indicated that all mothers had adequate milk supply [22]. Feeding and sleep episodes occurred frequently across the circadian cycle, consistent with infant behaviour during this developmental stage [3,6,23]. Considerable within-infant variability was observed across successive feeding and sleep episodes, including variation in feed volume, energy intake, and sleep duration [2]. This within-infant variability reflects rapid developmental change and sensitivity to environmental and circadian cues, highlighting the importance of time-ordered analyses when examining relationships between infant feeding and sleep.

We observed mean fat, protein, and lactose concentrations consistent with published reference ranges for mature breast milk [8,9] (Table 3). As expected, human milk fat contributed the largest proportion (~50%) of total breast milk energy [8,24]. Fat is the most variable macronutrient of human milk; post-feed milk may contain two to three times more fat than pre-feed milk; hence, the importance of utilising paired pre- and post-feed fat measurements in this study to calculate accurate feed-level energy intake [11]. In contrast, lactose and protein concentrations are much less variable over 24 h and reflect the established macronutrient hierarchy of human milk [8,10,25]. Consistent with this variability, descriptive 24-h profiles showed fluctuations in fat, protein, lactose and energy intake per breastfeeding episode (Figure 2). Leghi et al. reported no significant differences in protein or lactose concentrations according to time of day, day of collection, or breast across a three-week sampling period in mature human milk, supporting the use of representative milk samples for estimating average macronutrient composition [26]. Total daily energy intake and energy density fell within expected physiological ranges for fully breastfed infants of this age [10,15]. These findings support the validity of the feed-level energy estimates used to examine associations with infant sleep behaviour.

Across all three mixed-effects models, breast milk energy was not associated with subsequent infant sleep duration (Table 4). Energy from the immediately preceding feed showed no relationship with sleep length, nor did energy from the two prior feeds or cumulative energy across three feeds. Additionally, these findings were further supported by the analysis of complete within-subject 24-h profiles, in which hourly energy, macronutrient and milk volume intakes were not associated with hourly sleep duration (Table 5). This indicates that the absence of an association was not limited to analyses linking individual feeding episodes with subsequent sleep but was also observed when nutritional intake and sleep were evaluated across the complete 24-h period. These findings indicate that the natural variation in breast milk energy intake was not associated with the duration of the subsequent sleep episode in healthy breastfed infants of mothers with adequate milk supply. This analysis addresses an important evidence gap. Previous research examining infant sleep in the context of feeding has largely focused on feeding method, feeding frequency, and feeding timing, rather than the energetic properties of individual breast milk feeds [7,23,27,28]. Where energy has been considered, studies have typically examined infant energy expenditure or differences between breastfeeding versus commercial milk formula, rather than feed-level energy intake [14,29]. Emerging work examining chronobiological signals further highlights that mechanisms beyond energy such as light–dark cycles and hormonal rhythms, may be important in regulating infant sleep–wake behaviour [3,5,30,31]. Although higher-energy feeds are often assumed to promote longer sleep, our findings do not support this assumption [4].

Breast milk energy was not associated with the time until the next feeding episode (Table 6). Higher-energy feeds were not associated with a longer interval to the next feed, suggesting that short-term variation in feed energy intake was not associated with subsequent feeding intervals in this cohort. Breastfed infants regulate intake across successive feeds, and both environmental and behavioural factors influence hunger cues. Gastric emptying studies in preterm infants have shown that larger feed volumes do not stay in the infant’s stomach longer than smaller feed volumes, gastric emptying occurs proportionally across different feed volumes [17]. Additionally, in term breastfed infants, concentrations of adiponectin, whey protein and casein:whey ratio have been associated with gastric emptying, and larger feed volumes have been associated with faster gastric emptying rates [19]. Previous work has shown that feeding frequency in early infancy reflects infant-driven demand and developmental maturation rather than fixed caloric thresholds [14,15]. Together, these findings indicate that increasing feed energy is not associated with a delay until the next feeding episode in healthy breastfed infants. These results challenge the common assumption that higher-energy or larger feeds will reduce night waking, supporting responsive feeding guidance based on infant cues rather than attempts to manipulate feed energy content.

This study has implications for both clinical practice and parental guidance. In healthy breastfed infants, variation in the energy content of individual breast milk feeds was not associated with the duration of the subsequent sleep episode. These findings do not support an association between short-term variation in breast milk energy intake and subsequent infant sleep duration. Other factors, including circadian and developmental processes, may contribute to infant sleep behaviour [32]. Strategies aimed at increasing infant satiety through feeding manipulations, such as larger feed volumes, dream feeds or prolonged feeding duration, may therefore have limited influence on subsequent infant sleep duration. Clinical guidance would benefit from emphasis on biological timing, consistent light–dark exposure, responsive feeding, and the normal developmental nature of night waking [33]. From a parental perspective, these findings challenge the common assumption that larger or more energy-dense feeds lead to longer sleep [4]. Supporting parents with evidence-based education on normal infant sleep patterns and circadian maturation may reduce anxiety and discourage unnecessary feeding interventions.

Strengths of this study include the 24-h intensive sampling design, objective measurement of every feed and biochemical quantification of macronutrient composition. Additionally, multiple continuous-time mixed-effects models were used to examine short-term temporal relationships between feeding and sleep. This design allows precise investigation of physiological effects within infants. Limitations include the cross-sectional design and modest cohort size, which may have limited the detection of small effects, as reflected by the relatively wide confidence intervals. The exclusion of dyads with low milk supply or formula supplementation and those without samples available for macronutrient analysis may have introduced selection bias and limits generalisability beyond exclusively breastfeeding dyads with adequate milk supply. Feeding and sleep were assessed over a single 24-h period, with sleep being parent-reported, and unmeasured factors such as household routines, maternal sleep and infant temperament may have contributed to residual variability. The interval between the preceding recorded feed and subsequent sleep onset was variable, potentially reducing the biological relevance of some feed–sleep pairings; however, restricting the analysis to intervals ≤4 h did not materially alter the results. Finally, feed-specific energy intake used participant-specific protein and lactose concentrations rather than feed-specific measurements, which may have attenuated small associations towards the null. Despite these limitations, the consistency of findings across the complementary analyses provides evidence that short-term variation in breast milk energy intake is not a major determinant of subsequent sleep duration or feeding intervals in this cohort.

## 4. Methods

### 4.1. Participants

**Figure 3:**
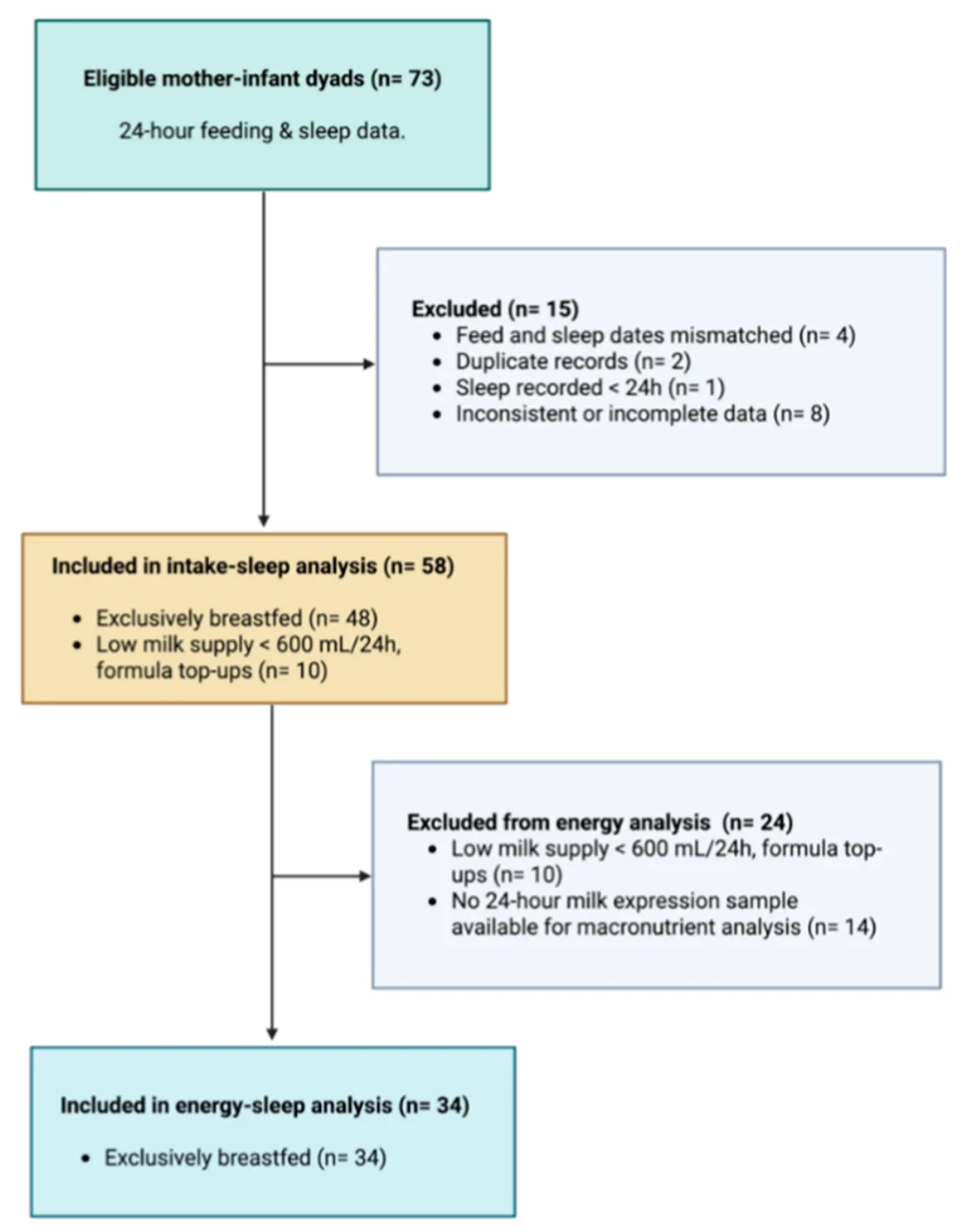
Flow diagram of participant selection for the energy–sleep analyses.

A prospective observational study was conducted using a within-subject, repeated-measures design. Breastfeeding women were recruited from the Western Australian community between 2022 and 2024. Participants with healthy term infants and no major pregnancy complications who were fully breastfeeding were included. Exclusion criteria included maternal smoking and preterm birth, and low milk supply requiring regular commercial milk formula supplementation. The study was conducted in accordance with the Declaration of Helsinki and approved by The University of Western Australia (UWA) Human Research Ethics Committee: 24 Hour Milk Profile Study, 2025/ET000355 (supersedes 2019/RA/4/6134; approved 28 April 2025), and Milk Composition Analysis, 2025/ET000838 (supersedes 2019/RA/4/20/6498; approved 23 March 2026). All participants provided written informed consent. A total of 58 participants provided 24-h data. Of these, 24 were excluded from the energy–sleep analysis: 10 due to low milk supply (<600 mL/24 h) and/or formula supplementation, and 14 because a participant-specific reference sample was unavailable for macronutrient analysis. The final energy–sleep analysis therefore included 34 exclusively breastfeeding mother–infant dyads (Figure 3).

### 4.2. Sample and Data Collection

Participants recorded 24-h infant feeding and sleep data, including pre- and post-feed weights, in their homes. Study data (sleep and feeding logs) were collected by mothers and managed by research staff using Research Electronic Data Capture (REDCap) tools [34,35].

Participant-specific reference breast milk samples used for macronutrient and energy composition analysis consisted of 5 mL aliquots obtained from a single full breast expression. These samples were collected by manual expression into 5 mL polypropylene tubes (P5016SL, Techno Plas Pty Ltd., St Marys, SA, Australia) during a supervised pumping session with the research team, within approximately 14 days of the 24-h feeding and sleep recording period. Collection was not standardised to a specific time of day or relative to infant feeding, as the purpose of the reference sample was to estimate each participant’s average milk composition rather than feed-specific composition. This approach was used to provide an estimate of each participant’s average milk composition [9,16]. Feed-specific fat concentrations used to calculate milk energy intake were derived from paired pre- and post-feed creamatocrit measurements collected during the 24-h study period, thereby accounting for within-feed variation in milk fat. The reference sample was therefore used to determine protein and lactose concentrations, which exhibit less short-term variation than fat. This approach is supported by evidence demonstrating that protein and lactose concentrations remain stable across the day and over several weeks in mature human milk, making a single representative sample appropriate for estimating average macronutrient composition [26]. These samples were stored at −80 °C until biochemical analysis.

Separately, mothers collected breast milk samples over the 24-h study period by expressing approximately 5 mL of milk (5 mL polypropylene tubes) immediately before and after each breastfeeding episode to obtain paired pre- and post-feed samples. Samples were stored immediately after expression in participants’ home freezers (typically −18 °C) and maintained under these conditions throughout the collection period, before transportation under chilled conditions to the UWA research laboratory. Upon arrival, pre-feed and post-feed samples were analysed for milk fat concentration at the individual feed level, before long-term storage at −80 °C.

### 4.3. Infant Intake Measurement

Infant breast milk intake was calculated using the test-weigh method [36]. Mothers were instructed to weigh the infant before and after feeding using a calibrated digital infant scale (±2.0 g; Electronic Baby Weigh Scale, Medela Inc., McHenry, IL, USA). The start and end times of each feed, along with the pre- and post-feed weights, were recorded on participant log-sheets. Participant data were then transcribed by the research team into REDCap and feed intake was calculated as *Intake (mL) = post-feed weight (g) − pre-feed weight (g)*, where 1 g of infant weight gain was considered equivalent to 1 mL (1.03 g) of breast milk intake [37].

### 4.4. Infant Sleep Recording

Participants recorded their infant’s sleep for the same 24-h period using a sleep diary, documenting each sleep period, sleep onset and wake times. The data entry of sleep logs was completed by research staff, who verified accuracy against the original maternal logs and recorded times using a 24-h clock before merging the datasets for analysis. Sleep duration was calculated in decimal hours.

### 4.5. Macronutrient Measurements

For macronutrient analysis, 1 mL of each milk sample was thawed in a 37 °C incubator for 30 min. The milk fat concentration was calculated using the creamatocrit method [38]. The fat concentration was measured in duplicate capillary tubes after centrifugation at 12,000× *g* for 10 min at room temperature. The mean percentage of the duplicates was used to calculate the concentration in relation to the cream layer and capillary height using the following equation [39]:

*Total fat concentration (g/L) = (creamatocrit [%] − 0.59)/0.146.*

The fat layer was then removed from the capillary and skimmed milk samples were collected for protein and lactose analysis. The protein concentration in the skim milk was quantified using the Bio-Rad DC Protein Assay Kit (modified Lowry method) [40], with human milk protein standards prepared according to the Kjeldahl method [41]. Lactose concentration was quantified using a colorimetric enzymatic assay (Megazyme Lactose Kit) [42]. Assay performance was monitored using pooled human milk quality control (QC) samples included on each plate. All samples were analysed in duplicate and the coefficient of variation (CV) between duplicate measurements was calculated for each sample. If the duplicate measurements exceeded a CV of 10%, the sample was reanalysed in duplicate until an acceptable value (CV < 10%) was achieved. This quality control procedure was implemented to ensure the precision and reliability of the macronutrient measurements. Final inter-assay precision was 3.9% for fat, 4.2% for protein, and 3.0% for lactose, with calibration curves demonstrating strong linearity across plates (R^2^ > 0.98). Results were attained for each macronutrient in g/L units.

### 4.6. Calculation of Total Energy, Total Macronutrient Intake and Energy Ratios

The estimated total energy concentration was calculated from the fat, protein and lactose assays using the following equation [43]:

*Total energy concentration (kcal/L) = Total fat concentration (g/L) × 9 (kcal/g) + Total protein concentration (g/L) × 4 (kcal/g) + Total lactose concentration (g/L) × 4 (kcal/g).*

The estimated 24-h fat, protein and lactose intakes per infant were calculated based on the following equation [18,44]:

*Total Intake (g/day) = Total concentration (g/L) × 24-h feed intake (L/day).*

Total energy intake was calculated using the equation below:

*Total energy intake (kcal/day) = Total energy concentration (kcal/L) × 24-h feed intake (L/day).*

Macronutrient energy ratios were calculated using the equation below [10]:

*% Energy = (Total macronutrient concentration [g/L] × representative kcal)/Total energy concentration [kcal/L]) × 100.*

### 4.7. Feed-Specific Breast Milk Macronutrient Concentration and Energy Intake

Protein and lactose intakes were calculated for each feed from the participant-specific reference breast milk concentrations using the equation below:

*Protein or lactose intake (g/feed) = (Macronutrient concentration [g/L] × milk feed intake [L]).*

Fat intake was calculated for each feed from the pre- and post-creamatocrit values using the equation below. Where these values were not recorded, the participant-specific reference fat assay concentration was used instead:

*Fat intake (g/feed) = (Pre-post-creamatocrit % average − 0.59/0.146) × (milk feed intake [L]).*

Energy intake per feed was then calculated using Atwater factors and the following equation:

*Energy intake (kcal/feed) = Fat concentration (g/feed) × 9 (kcal/g) + Protein concentration (g/feed) × 4 (kcal/g) + Lactose concentration (g/feed) × 4 (kcal/g).*

The milk samples and data sources used for each derived macronutrient and energy calculation are summarised in Supplementary Table S3.

### 4.8. Statistical Analysis

Statistical analyses were performed in R version 4.5.2 (R Foundation for Statistical Computing, Vienna, Austria) using RStudio. Descriptive statistics are reported as mean ± standard deviation (SD) for continuous variables and frequencies and percentages for categorical variables. Normality of continuous variables was assessed using the Shapiro–Wilk test. Feeding and sleep data were imported from Excel and structured at the episode level. Date-time variables were converted to a 24-h time format and sleep episodes were classified as daytime (06:00–17:59) or nighttime (18:00–05:59) according to the clock time of infant sleep onset. These fixed 12-h intervals were selected to provide a broad and standardised classification of day and night for inclusion as a covariate in the statistical models. The classification was based on clock time and was not intended to correspond precisely to seasonal photoperiod or individual light exposure.

For each sleep episode, the most recent feeding episode preceding sleep onset was identified using time-stamped records. Only sleep episodes with an identifiable preceding feed were included in the analytical dataset. Of the 249 valid sleep episodes recorded, 237 had an identifiable preceding feed and were retained for analysis. Twelve episodes (4.8%) were excluded because no preceding feed could be identified from the time-stamped feeding records. Feed-level energy intake (kcal) was calculated from macronutrient intakes using Atwater conversion factors.

Linear mixed-effects models were fitted using the *lme4* package (lmer function) to examine associations between feed energy intake and subsequent sleep duration, with infant ID included as a random intercept to account for repeated measures within infants. Three complementary model structures were used to examine potential immediate, delayed and cumulative associations: (1) a single-feed model including energy intake from the immediately preceding feed; (2) a distributed lag model including energy intake from the most recent feed (t) and the two preceding feeds (t^−1^, t^−2^); and (3) a cumulative model including summed energy intake across the three preceding feeds. Energy variables were scaled by 10 kcal to improve interpretability. Models were adjusted for infant age (months), time of day (day vs. night), birth weight (grams), and 24-h breast milk production (mL). Temporal autocorrelation between successive sleep episodes was accounted for using a continuous-time first-order autoregressive [AR(1)] correlation structure using the *nlme* package, based on sleep onset time.

To additionally examine the relationship between nutritional intake and sleep across the complete 24-h period, a within-subject hourly time-series analysis was conducted. For each infant, the 24-h recording period was divided into 24 hourly intervals. Milk volume and corresponding energy, fat, protein and lactose intakes from all feeding episodes occurring within each interval were aggregated to derive hourly intake measures, while sleep was quantified as the total number of minutes asleep within each hourly interval. Linear mixed-effects models were fitted for each nutritional exposure, with infant ID included as a random intercept. An AR(1) correlation structure was used to account for temporal correlation between adjacent hourly observations. Models were adjusted for infant age, day/night period, birth weight and 24-h milk production.

Time to the next feeding episode was analysed using the same mixed-effects framework. Inter-feed intervals were defined as the elapsed time from the end of each breastfeeding episode to the start of the subsequent breastfeeding episode.

Multicollinearity among the fixed-effects covariates was assessed using variance inflation factors (VIFs). Obtained values of <1.14 for all models was an indicator of no multicollinearity. A sensitivity analysis was conducted by repeating the three mixed-effects models after excluding infants younger than 2 months and older than 6 months to evaluate whether the inclusion of infants at the extremes of the age range influenced the findings. Additionally, a post hoc sensitivity analysis was also conducted restricting the primary single-feed model to sleep episodes occurring within 4 h of the preceding feed to assess the robustness of the findings to more temporally distant feed–sleep pairings.

Model fit was assessed using the Akaike Information Criterion (AIC), and model assumptions were assessed by visual inspection of residuals versus fitted values and normal quantile–quantile (Q–Q) plots of the normalised residuals. Visual inspection of the residual diagnostic plots indicated no substantial violations of model assumptions. Minor departures from normality were evident in the upper tail of the residual distribution, but these were consistent across models and were not considered sufficient to affect model interpretation (Supplementary Figure S1). Results are reported as regression coefficients (*β*), 95% confidence intervals (CI), and *p*-values, with statistical significance defined as *p* < 0.05.

The authors used ChatGPT 5.2 (OpenAI) to assist with R code formatting and troubleshooting. The authors take full responsibility for the analysis and interpretation of results. This study was reported in accordance with the STROBE-nut reporting guidelines for nutritional epidemiology [45].

## 5. Conclusions

This study examined whether breast milk energy intake was associated with subsequent infant sleep duration and time until the next feed using 24-h, feed-level measurements in healthy, exclusively breastfed infants. Energy intake from the immediately preceding feed was not significantly associated with subsequent sleep duration, and no associations were observed for lagged or cumulative energy intake. Similarly, energy intake was not associated with the time until the next breastfeed. The within-subject hourly analyses further demonstrated no significant associations between energy, fat, protein or lactose intake and sleep duration across the 24-h period.

These findings do not support an association between short-term variation in breast milk energy intake and infant sleep or feeding behaviour in this cohort. Although breast milk composition and intake varied across the day, this variation did not explain differences in subsequent sleep duration or feeding intervals. Other biological and developmental factors, including circadian processes, may contribute to these behaviours. The findings challenge common assumptions that higher-energy feeds are associated with longer infant sleep and support guidance emphasising responsive feeding and realistic expectations of infant sleep development. Further research incorporating larger longitudinal cohorts, objective sleep measurements and additional biological factors is warranted to better understand the mechanisms regulating infant sleep–wake behaviour.

## Figures and Tables

**Table 1:** Maternal and infant characteristics (*n* = 34).

Characteristics	*n* (%) or Mean ± SD
Maternal Age (years)	34.1 ± 4.7
Parity	
0	20 (58.8%)
1	9 (26.5%)
2+	5 (14.7%)
Pre-Pregnancy BMI (kg/m^2^)	26.5 ± 4.8
Ethnicity	
Australian	27 (79.4%)
Asian	1 (2.9%)
British	8 (23.5%)
South-East European	1 (2.9%)
Central or South American	1 (2.9%)
Birth gestation (weeks)	39.5 ± 1.4
Birth weight (grams)	3482.1 ± 384.6
Infant Sex	
Female	19 (55.9%)

Note: Values are presented as mean ± standard deviation or number %. BMI = body mass index. Participants could select multiple ethnicities. 2+ refers to more than two children.

**Table 2:** Twenty-four-hour descriptive characteristics of the study cohort (*n* = 34).

Variable	Mean ± SD (Range)
Infant age at sampling (months)	3.7 ± 1.3 (1.0–6.7)
Total time monitored (hours)	26.1 ± 2.0 (23.6–32.2)
Total 24-h infant intake (mL)	851.8 ± 194.2 (602–1148)
Breastfeeds	16.1 ± 4.9 (8–28)
Inter-feed Intervals (min)	104 ± 84.9 (2–335)
Total 24-h breast milk production (mL)	873.2 ± 191.2 (627.5–1113.8)
Total 24-h sleep duration (hours)	15.5 ± 3.4 (10.9–23.3)
Sleep episodes	7.2 ± 1.6 (5–9)

Note: Values are presented as mean ± standard deviation or number %, with ranges provided where relevant. Elapsed time represents the total duration of concurrent feeding and sleep recording. Total intake is the cumulative breast milk volume consumed by the infant; breast milk production is the total maternal milk breastfed and expressed over a 24-h period; inter-feed interval is the time between breastfeeding episodes.

**Table 3:** Breast milk macronutrient concentrations and intakes (*n* = 34).

Macronutrient	Concentration (g/L or kcal/L)	Intake (g/day or kcal/Day)
Fat	38.5 ± 15.4	32.5 ± 14.5
Protein	9.1 ± 3.1	7.6 ± 2.5
Lactose	73.0 ± 15.2	61.9 ± 17.9
Energy	674.7 ± 140.7	570.8 ± 154.0
Fat:energy ratio	-	49.8 ± 12.1%
Protein:energy ratio	-	5.6 ± 2.2%
Lactose:energy ratio	-	44.6 ± 11.4%

Note: Values are presented as mean ± standard deviation or number %. Energy intake and macronutrient-derived energy ratios were calculated using Atwater conversion factors (fat: 9 kcal/g; protein and lactose: 4 kcal/g). Participant-specific reference breast milk samples were used for macronutrient and energy composition analysis, consisting of 5 mL aliquots obtained from a single full breast expression within approximately 14 days of the 24-h feeding and sleep recording period.

**Table 4:** Associations between infant breast milk energy intake and subsequent sleep duration (*n* = 34).

Predictor	Effect Size (*β*, min)	95% CI	*p*-Value
**Model 1**			
Energy (per 10 kcal)	4.13	−0.89 to 9.15	0.106
Infant age (months)	−5.75	−18.64 to 7.14	0.370
Night vs. Day	0.52	−31.70 to 32.74	0.975
Birth weight (grams)	−0.022	−0.021 to 0.065	0.302
Milk production (mL/24 h)	0.0025	−0.084 to 0.090	0.953
**Model 2**			
Energy_t (per 10 kcal)	3.52	−1.53 to 8.58	0.171
Energy_t^−1^	2.28	−3.19 to 7.74	0.412
Energy_t^−2^	−1.87	−7.57 to 3.82	0.518
Infant age (months)	−7.06	−19.49 to 5.37	0.255
Night vs. Day	−5.15	−36.44 to 26.15	0.746
Birth weight (grams)	0.019	−0.022 to 0.061	0.347
Milk production (mL/24 h)	−0.012	−0.0997 to 0.0752	0.776
**Model 3**			
Cumulative energy*	1.37	−1.18 to 3.91	0.290
Infant age (months)	−6.23	−18.56 to 6.11	0.311
Night vs. Day	−6.26	−37.47 to 24.96	0.693
Birth weight (grams)	0.016	−0.025 to 0.057	0.430
Milk production (mL/24 h)	−0.011	−0.0977 to 0.0755	0.795

Note: Effect sizes (*β*) represent the estimated change in infant sleep duration (minutes). Energy variables were scaled per 10 kcal to aid interpretability. Energy (t) represents the energy content (kcal) of the immediately preceding feed prior to sleep onset; Energy (t^−1^) and Energy (t^−2^) represent the energy content of the second and third most recent feeds, respectively. Cumulative energy* represents the sum of energy intake across the three most recent feeds (t + t^−1^ + t^−2^). Continuous covariates (infant age, birth weight, and 24 h milk production) reflect the expected minute change in sleep duration per one-unit increase in the predictor. For the categorical variable Night vs. Day, *β* represents the mean difference in sleep duration between nighttime and daytime sleep episodes. Three mixed-effects models were fitted using a continuous-time first-order autoregressive [AR(1)] correlation structure to account for temporal dependence between successive sleep episodes within infants. Model 1 examined the association between sleep duration and energy intake from the immediately preceding feed only (*n* = 237 sleep episodes; AIC = 2957). Model 2 assessed distributed lag effects including energy intake from the preceding feed and the two prior feeds (t, t^−1^, t^−2^; *n* = 232 sleep episodes; AIC = 2869). Model 3 evaluated cumulative energy intake across the three most recent feeds (*n* = 232 sleep episodes; AIC = 2876). All models included infant ID as a random intercept and were adjusted for infant age, time of day (day vs. night), birth weight, and 24 h milk production.

**Table 5:** Within-subject 24-h associations between breast milk and macronutrient intake and infant sleep duration.

Predictor	Effect Size (*β*, min)	95% CI	*p*-Value
Energy, per 10 kcal	−0.152	−0.433 to 0.130	0.290
Fat, per 1 g	−0.274	−0.726 to 0.178	0.234
Protein, per 1 g	−1.124	−3.249 to 1.002	0.300
Lactose, per 1 g	−0.117	−0.395 to 0.161	0.407
Milk Volume, per 100 mL	−1.044	−3.014 to 0.926	0.299

Note: Effect sizes (*β*) represent the estimated difference in minutes of sleep per hourly increment in the specified predictor. Model included 816 hourly observations from 34 infants and were adjusted for by infant age, day/night period, birth weight and 24-h milk production. A random infant intercept and an AR(1) correlation structure were included to account for repeated within-infant observations and temporal autocorrelation.

**Table 6:** Association between breast milk energy and time until next feed (*n* = 34).

Predictor	Effect Size (*β*, %)	95% CI	*p*-Value
Energy (per 10 kcal)	−2.4	−6.2 to 1.4	0.212
Infant age (months)	8.1	−5.0 to 22.8	0.233
Night vs. Day	−3.4	−24.6 to 23.4	0.774
Birth weight (grams)	<0.1	−0.4 to 0.5	0.798
Milk production (mL/24 h)	<0.1	−0.8 to 0.9	0.923

Note: Effect sizes represent percent change in time until the next feed derived from log-transformed linear mixed-effects models. Energy was scaled per 10 kcal. Models included infant-level random intercepts and a continuous autoregressive correlation structure to account for repeated measures and temporal autocorrelation.

## Data Availability

Restrictions apply to the availability of some, or all data generated or analysed during this study. The corresponding author, will, on request, detail the restrictions and any conditions under which access to some data may be provided.

## Supplementary Materials

The following supporting information can be downloaded at: https://www.mdpi.com/article/10.3390/clockssleep8030056/s1, Table S1: Comparison of unadjusted and adjusted associations between breast milk energy intake and infant sleep and feeding outcomes; Table S2: Sensitivity analysis of the association between feed energy intake and subsequent infant sleep duration after excluding infants younger than 2 months and older than 6 months; Table S3: Milk samples used for each calculation; Figure S1: Residual diagnostic plots for the three linear-mixed-effects models.

## Abbreviations

AIC, Akaike Information Criterion; AR(1), autoregressive (1); α, significance level; *β*, regression coefficient; BMI, body mass index; CI, confidence interval; CV, coefficient of variation; ICC, intraclass correlation coefficient; IQR, interquartile range; LMS, low milk supply; *p*, probability value; Q-Q, quantile–quantile; *r*, Pearson correlation coefficient; SE, standard error; VIF, variance inflation factor.

## References

[1] Galland B.C., Taylor B.J., Elder D.E., Herbison P. (2012). Normal sleep patterns in infants and children: A systematic review of observational studies. Sleep Med. Rev..

[2] Löhr B., Siegmund R. (1999). Ultradian and Orcadian Rhythms of Sleep-Wake and Food-Intake Behavior During Early Infancy. Chronobiol. Int..

[3] Rivkees S.A. (2003). Developing Circadian Rhythmicity in Infants. Pediatrics.

[4] Perrella S.L., Dix-Matthews A., Williams J., Rea A., Geddes D.T. (2022). Breastfeeding and Maternal Perceptions of Infant Sleep, Settle and Cry Patterns in the First 9 Months. Int. J. Environ. Res. Public Health.

[5] Engler A.C., Hadash A., Shehadeh N., Pillar G. (2012). Breastfeeding may improve nocturnal sleep and reduce infantile colic: Potential role of breast milk melatonin. Eur. J. Pediatr..

[6] Iglowstein I., Jenni O.G., Molinari L., Largo R.H. (2003). Sleep Duration from Infancy to Adolescence: Reference Values and Generational Trends. Pediatrics.

[7] Quillin I.M., Glenn L.L. (2004). Interaction Between Feeding Method and Co-Sleeping on Maternal-Newborn Sleep. J. Obstet. Gynecol. Neonatal Nurs..

[8] Ballard O., Morrow A.L. (2013). Human milk composition: Nutrients and bioactive factors. Pediatr. Clin. N. Am..

[9] Mitoulas L.R., Kent J.C., Cox D.B., Owens R.A., Sherriff J.L., Hartmann P.E. (2002). Variation in fat, lactose and protein in human milk over 24 h and throughout the first year of lactation. Br. J. Nutr..

[10] Ma J., Palmer D.J., Lai C.T., Prescott S.L., D’Vaz N., Vlaskovsky P., Stinson L.F., Gridneva Z., Geddes D.T. (2024). Macronutrients in Human Milk and Early Childhood Growth—Is Protein the Main Driver?. Nutrients.

[11] Saarela T., Kokkonen J., Koivisto M. (2005). Macronutrient and energy contents of human milk fractions during the first six months of lactation. Acta Paediatr..

[12] George A.D., Gay M.C.L., Wlodek M.E., Geddes D.T. (2021). The importance of infants’ lipid intake in human milk research. Nutr. Rev..

[13] Norrish I., Sindi A., Sakalidis V.S., Lai C.T., McEachran J.L., Tint M.T., Perrella S.L., Nicol M.P., Gridneva Z., Geddes D.T. (2023). Relationships between the Intakes of Human Milk Components and Body Composition of Breastfed Infants: A Systematic Review. Nutrients.

[14] Butte N.F., Jensen C.L., Moon J.K., Glaze D.G., Frost J.D. (1992). Sleep Organization and Energy Expenditure of Breast-Fed and Formula-Fed Infants. Pediatr. Res..

[15] Dewey K.G., Heinig M.J., Nommsen L.A., Peerson J.M., Lonnerdal B. (1992). Growth of breast-fed and formula-fed infants from 0 to 18 months: The DARLING Study. Pediatrics.

[16] Kent J.C., Mitoulas L.R., Cregan M.D., Ramsay D.T., Doherty D.A., Hartmann P.E. (2006). Volume and Frequency of Breastfeedings and Fat Content of Breast Milk Throughout the Day. Pediatrics.

[17] Perrella S.L., Hepworth A.R., Gridneva Z., Simmer K.N., Hartmann P.E., Geddes D.T. (2018). Gastric emptying of different meal volumes of identical composition in preterm infants: A time series analysis. Pediatr. Res..

[18] Suwaydi M.A., Lai C.T., Gridneva Z., Perrella S.L., Wlodek M.E., Geddes D.T. (2024). Sampling Procedures for Estimating the Infant Intake of Human Milk Leptin, Adiponectin, Insulin, Glucose, and Total Lipid. Nutrients.

[19] Gridneva Z., Kugananthan S., Hepworth A.R., Tie W.J., Lai C.T., Ward L.C., Hartmann P.E., Geddes D.T. (2016). Effect of Human Milk Appetite Hormones, Macronutrients, and Infant Characteristics on Gastric Emptying and Breastfeeding Patterns of Term Fully Breastfed Infants. Nutrients.

[20] Nommsen-Rivers L.A., Wagner E.A., Roznowski D.M., Riddle S.W., Ward L.P., Thompson A. (2022). Measures of Maternal Metabolic Health as Predictors of Severely Low Milk Production. Breastfeed. Med..

[21] Davis K.F., Parker K.P., Montgomery G.L. (2004). Sleep in infants and young children: Part one: Normal sleep. J. Pediatr. Health Care.

[22] Kent J.C., Mitoulas L., Cox D.B., Owens R.A., Hartmann P.E. (1999). Breast Volume and Milk Production During Extended Lactation in Women. Exp. Physiol..

[23] Tikotzky L., Sadeh A. (2009). Maternal Sleep-Related Cognitions and Infant Sleep: A Longitudinal Study from Pregnancy through the 1st Year. Child Dev..

[24] Butte N.F., Fox M.K., Briefel R.R., Siega-Riz A.M., Dwyer J.T., Deming D.M., Reidy K.C. (2010). Nutrient Intakes of US Infants, Toddlers, and Preschoolers Meet or Exceed Dietary Reference Intakes. J. Am. Diet. Assoc..

[25] Hester S.N., Hustead D.S., Mackey A.D., Singhal A., Marriage B.J. (2012). Is the Macronutrient Intake of Formula-Fed Infants Greater Than Breast-Fed Infants in Early Infancy?. J. Nutr. Metab..

[26] Leghi G.E., Lai C.T., Narayanan A., Netting M.J., Dymock M., Rea A., Wlodek M.E., Geddes D.T., Muhlhäusler B.S. (2021). Daily variation of macronutrient concentrations in mature human milk over 3 weeks. Sci. Rep..

[27] Mindell J.A., Leichman E.S., DuMond C., Sadeh A. (2017). Sleep and Social-Emotional Development in Infants and Toddlers. J. Clin. Child Adolesc. Psychol..

[28] Wang W., Huang L., Zhang X., Lin L., Chen X., Zhong C., Chen R., Wu M., Yang S., Tu M. (2023). Association of Breastfeeding Practices During the First 3 Months with Infant Sleep Trajectories: A Prospective Cohort Study. J. Nutr..

[29] Wells J.C., Cole T.J., Davies P.S. (1996). Total energy expenditure and body composition in early infancy. Arch. Dis. Child..

[30] Blumberg M.S., Gall A.J., Todd W.D. (2014). The Development of Sleep-Wake Rhythms and the Search for Elemental Circuits in the Infant Brain. Behav. Neurosci..

[31] Caba-Flores M.D., Ramos-Ligonio A., Camacho-Morales A., Martínez-Valenzuela C., Viveros-Contreras R., Caba M. (2022). Breast Milk and the Importance of Chrononutrition. Front. Nutr..

[32] Douglas P.S., Hill P.S. (2013). Behavioral Sleep Interventions in the First Six Months of Life Do not Improve Outcomes for Mothers or Infants: A Systematic Review. J. Dev. Behav. Pediatr..

[33] McKenna H., Reiss I.K.M. (2018). The case for a chronobiological approach to neonatal care. Early Hum. Dev..

[34] Harris P.A., Taylor R., Thielke R., Payne J., Gonzalez N., Conde J.G. (2009). Research electronic data capture (REDCap)—A metadata-driven methodology and workflow process for providing translational research informatics support. J. Biomed. Inform..

[35] Harris P.A., Taylor R., Minor B.L., Elliott V., Fernandez M., O’Neal L., McLeod L., Delacqua G., Delacqua F., Kirby J. (2019). The REDCap consortium: Building an international community of software platform partners. J. Biomed. Inform..

[36] Kent J.C., Hepworth A.R., Langton D.B., Hartmann P.E. (2015). Impact of Measuring Milk Production by Test Weighing on Breastfeeding Confidence in Mothers of Term Infants. Breastfeed. Med..

[37] Neville M.C., Keller R., Seacat J., Lutes V., Lutes M., Casey C., Allen J., Archer P. (1988). Studies in human lactation: Milk volumes in lactating women during the onset of lactation and full lactation. Am. J. Clin. Nutr..

[38] Wang C.D., Chu P.S., Mellen B.G., Shenai J.P. (1999). Creamatocrit and the Nutrient Composition of Human Milk. J. Perinatol..

[39] Lucas A., Gibbs J.A., Lyster R.L., Baum J.D. (1978). Creamatocrit: Simple clinical technique for estimating fat concentration and energy value of human milk. BMJ.

[40] Lowry O.H., Rosebrough N.J., Farr A.L., Randall R.J. (1951). Protein measurement with the Folin phenol reagent. J. Biol. Chem..

[41] Atwood C.S., Hartmann P.E. (1992). Collection of fore and hind milk from the sow and the changes in milk composition during suckling. J. Dairy Res..

[42] Arthur P.G., Smith M., Hartmann P.E. (1989). Milk Lactose, Citrate, and Glucose as Markers of Lactogenesis in Normal and Diabetic Women. J. Pediatr. Gastroenterol. Nutr..

[43] Perrin M.T., Belfort M.B., Hagadorn J.I., McGrath J.M., Taylor S.N., Tosi L.M., Brownell E.A. (2020). The Nutritional Composition and Energy Content of Donor Human Milk: A Systematic Review. Adv. Nutr..

[44] EFSA Panel on Dietetic Products, Nutrition and Allergies (NDA) (2013). Scientific Opinion on nutrient requirements and dietary intakes of infants and young children in the European Union. EFSA J..

[45] Lachat C., Hawwash D., Ocké M.C., Berg C., Forsum E., Hörnell A., Larsson C., Sonestedt E., Wirfält E., Åkesson A. (2016). Strengthening the Reporting of Observational Studies in Epidemiology—Nutritional Epidemiology (STROBE-nut): An Extension of the STROBE Statement. PLoS Med..

