# Supplementary material for: Associations Between Breast Milk Energy Intake, Infant Sleep Duration and Time Until Next Feed
Source: Clocks Sleep. 2026 Sep 8;8(3):56. doi: 10.3390/clockssleep8030056 (PMC13605171; doi:10.3390/clockssleep8030056)

Supplementary Table S1: Comparison of unadjusted and adjusted associations between breast milk energy intake and infant sleep and feeding outcomes.

Analysis	Unadjusted β (95% CI)	Adjusted β (95% CI)
Model 1: Energy (per 10 kcal)	3.67 min (-1.23 to 8.57)	4.13 min (-0.89 to 9.15)
Model 2: Energy_t	2.94 min (-2.01 to 7.89)	3.52 min (-1.53 to 8.58)
Model 2: Energy_t^{-1}	2.13 min (-3.17 to 7.43)	2.28 min (-3.19 to 7.74)
Model 2: Energy_ t^{-2}	-1.38 min (-7.00 to 4.24)	-1.87 min (-7.57 to 3.82)
Model 3: Cumulative energy	1.29 min (-1.16 to 3.75)	1.37 min (-1.18 to 3.91)
Time to next feed	-2.25% (−5.91 to 1.55)	-2.4% (-6.2 to 1.4)

Supplementary Table S2. Sensitivity analysis of the association between feed energy intake and subsequent infant sleep duration after excluding infants younger than 2 months and older than 6 months.

Predictor	Effect Size (β, min)	95% CI	*p*-value
**Model 1**			
Energy (per 10 kcal)	3.60	-1.89 to 9.09	0.197
Infant age (months)	0.30	-24.53 to 25.13	0.980
Night vs. Day	-0.38	-37.08 to 36.32	0.984
Birth weight (grams)	0.014	-0.039 to 0.067	0.586
Milk production (mL/24 h)	0.017	-0.107 to 0.140	0.784
**Model 2**			
Energy_t (per 10 kcal)	2.81	-2.75 to 8.37	0.319
Energyt_t^{-1}	0.90	-5.06 to 6.86	0.766
Energyt^{-2}	−1.10	-7.52 to 5.32	0.735
Infant age (months)	−1.05	-24.93 to 22.83	0.929
Night vs. Day	−8.16	-43.96 to 27.65	0.653
Birth weight (grams)	0.014	-0.037 to 0.065	0.577
Milk production (mL/24 h)	−0.004	-0.127 to 0.119	0.951
**Model 3**			
Cumulative energy	0.955	-1.81 to 3.72	0.495
Infant age (months)	-0.065	-23.54 to 23.41	0.995
Night vs. Day	-9.70	-45.08 to 25.68	0.589
Birth weight (grams)	0.011	-0.039 to 0.061	0.653
Milk production (mL/24 h)	-0.005	-0.125 to 0.115	0.927

Note: In a sensitivity analysis excluding infants younger than 2 months or older than 6 months, 28 infants and 195 sleep episodes remained. The observed age range was 2.37-5.32 months. The findings were unchanged, with no evidence of associations between energy intake in the preceding feed, energy intake in earlier feeds, or cumulative energy intake and subsequent infant sleep duration.

Supplementary Table S3. Milk samples used for each calculation.

Calculation	Formula	Milk sample(s) used
Total energy concentration (kcal/L)	*Total energy concentration (kcal/L) = Total fat concentration (g/L) x 9 (kcal/g) + Total protein concentration (g/L) x 4 (kcal/g) + Total lactose concentration (g/L) x 4 (kcal/g)*	Participant-specific reference sample (fat, protein and lactose assays)
24-hour fat intake (g/day)	*Total Intake (g/day) = Fat concentration (g/L) x 24-hour feed intake (L/day)*	Participant-specific reference sample + 24-hour milk intake
24-hour protein intake (g/day)	*Total Intake (g/day) = Protein concentration (g/L) x 24-hour feed intake (L/day)*	Participant-specific reference sample + 24-hour milk intake
24-hour lactose intake (g/day)	*Total Intake (g/day) = Lactose concentration (g/L) x 24-hour feed intake (L/day)*	Participant-specific reference sample + 24-hour milk intake
24-hour energy intake (kcal/day)	*Total energy intake (kcal/day) = Total energy concentration (kcal/L) x 24-hour feed intake (L/day)*	Participant-specific reference sample + 24-hour milk intake
Macronutrient energy ratios (%)	*% Energy = (Total macronutrient concentration [g/L] x representative kcal) / Total energy concentration [kcal/L]) x 100*	Participant-specific reference sample
Protein intake (g/feed)	*Protein intake (g/feed) = (Macronutrient concentration [g/L] x feed intake [L])*	Participant-specific reference sample + feed-specific milk intake volume
Lactose intake (g/feed)	*Lactose intake (g/feed) = (Macronutrient concentration [g/L] x feed intake [L])*	Participant-specific reference sample + feed-specific milk intake volume
Fat intake (g/feed)	*Fat intake (g/feed) = (Pre- post creamatocrit % average – 0.59 / 0.146) x (feed intake volume [L])*	Pre- and post-feed creamatocrit (average) + feed-specific milk intake volume*
Energy intake (kcal/feed)	*Energy intake (kcal/feed) = Fat concentration (g/feed) x 9 (kcal/g) + Protein concentration (g/feed) x 4 (kcal/g) + Lactose concentration (g/feed) x 4 (kcal/g)*	Fat intake from pre-/post-feed creamatocrit*; protein and lactose intake from the participant-specific reference sample

Note: Where pre- or post-feed creamatocrit measurements were unavailable, fat concentration from the participant-specific reference sample was used*.

Supplementary Figure S1. Residual diagnostic plots for the three linear-mixed-effects models.

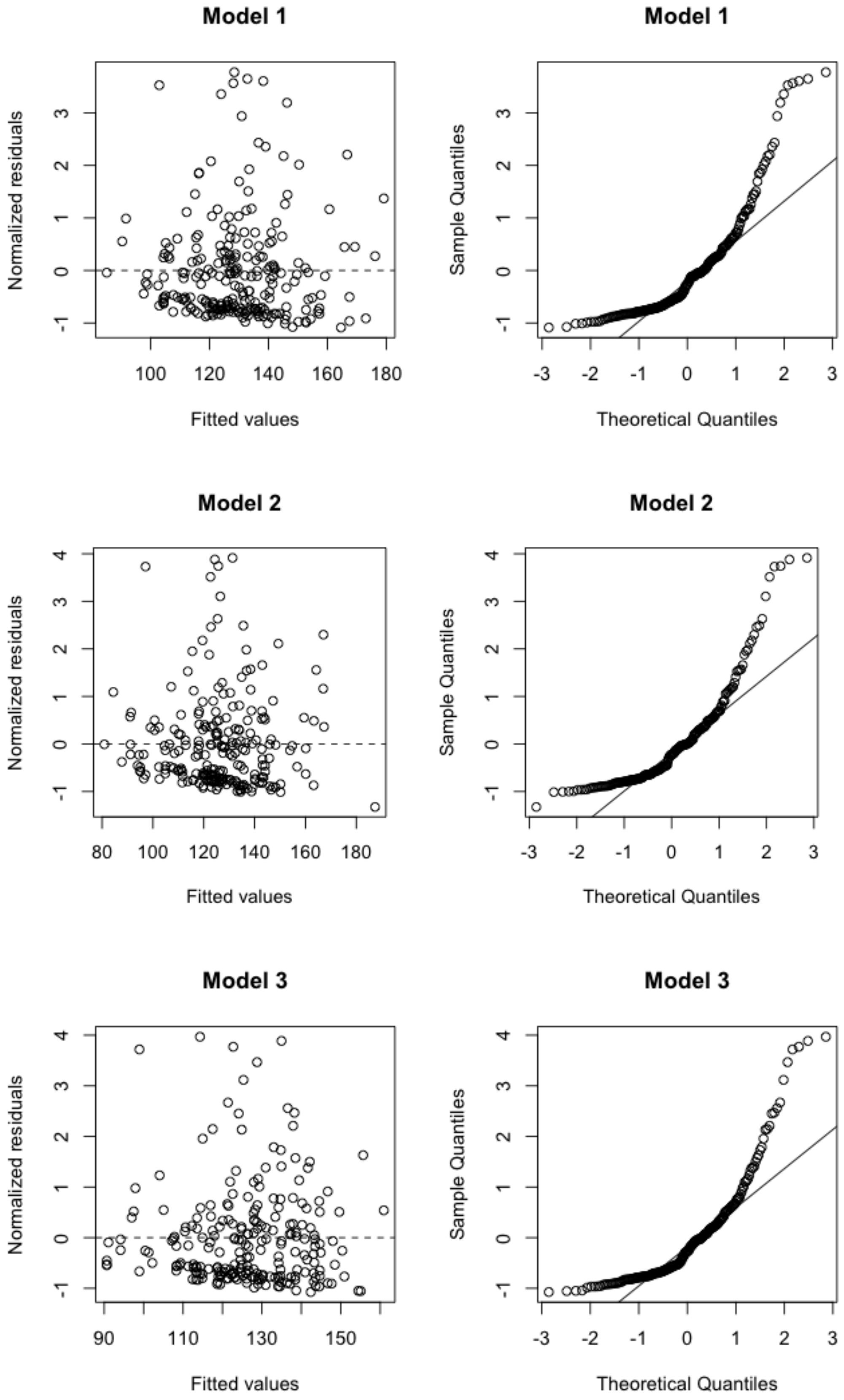

Supplement: Supplementary file 1 [file clockssleep-08-00056-s001.zip › clockssleep-4421352-supplementary.pdf]
